# Transmission of Ovine Herpesvirus 2 from Asymptomatic Boars to Sows

**DOI:** 10.3201/eid1612.101453

**Published:** 2010-12

**Authors:** Érica Azevedo Costa, Aline de Marco Viott, Glauber de Souza Machado, Maria Rosa Quaresma Bomfim, Fabiana Magalhães Coelho, Zélia Inês Portela Lobato, Mauricio Resende, Roberto Mauricio Carvalho Guedes

**Affiliations:** Author affiliations: Universidade Federal de Minas Gerais, Belo Horizonte, Minas Gerais, Brazil (É. Azevedo Costa, G. de Souza Machado, M.R. Quaresma Bomfim, F. Magalhães Coelho, Z.I. Portela Lobato, M. Resende, R.M. Carvalho Guedes);; Universidade Federal do Paraná, Palotina, Paraná, Brazil (A. de Marco Viott)

**Keywords:** Malignant catarrhal fever, swine, semen, Brazil, viruses, letter

**To the Editor:** Malignant catarrhal fever (MCF) is an often lethal viral disease of susceptible biungulates from the Bovidae, Cervidae, and Suidae subfamilies. MCF in pigs has been associated with direct or indirect contact with sheep, which are the main reservoir of ovine herpesvirus 2 (OvHV-2) (*1*). A recent report detected infected but asymptomatic swine in the absence of known exposure to sheep or goats (*2*). Porcine MCF is difficult to diagnose because of its nonspecific clinical signs and sporadic nature; however, an outbreak involving 41 swine has been described (*3*). Pigs are terminal hosts and are not believed to spread the virus. Here we describe OvHV-2 DNA in the blood and semen of asymptomatic boars and from the brain of symptomatic sows and gilts with MCF that was probably transmitted by artificial insemination.

The MCF cases occurred on two 3-site commercial farms with 2,700 and 1,670 sows in 2 different counties in southwestern Brazil. No MCF losses previously had been recorded in the region, and the animals had no known direct or indirect contact with sheep. The 2 farms had high biosecurity. The first case was recorded in September 2004, and the number of cases increased in July 2006. Twenty-eight sows and gilts, 20 of them pregnant and at >25 days’ gestation, died during January 2007–March 2008, when the last case was observed.

Clinical features in sows and gilts were depression followed by abortion, fever (41°C), and anorexia. After the onset of clinical signs, neurologic symptoms developed such as ataxia, tremors, convulsions, and aggressive behavior. Animals that survived longer showed forelimb paralysis, stood in a dog-sit position, and gnawed with abundant salivation on pen bars.

Specimens from randomly selected dead sows and gilts from the outbreaks during 2004–2008 were obtained for histopathologic examination, immunofluorescence testing for rabies virus, viral and bacterial isolation, and PCR. No bacterial or viral growth was detected, and direct immunofluorescence for rabies virus was negative. Microscopic examination showed high-grade nonpurulent meningoencephalitis characterized by lymphocytic cuffings with vasculitis in the brain hemisphere, the brainstem, the spinal cord, and, to a lesser extent, the cerebellum. Multifocal areas of edema, fibrinoid necrosis, and lymphocytic infiltration also were observed (Figure). OvHV-2 DNA was detected by using a specific PCR (*4*) in 5 of 7 paraffinized sections of the brainstem (*5*). To analyze the possible presence of other porcine lymphotropic herpesviruses in samples that reacted positively for OvHV-2, a nested PCR with degenerate primers (*6**,**7*) was applied. None of the OvHV-2–positive samples reacted positively for porcine lymphotropic herpesviruses. To confirm that the virus was a member of the MCFV group, we purified 1 amplicon and submitted it for automated sequencing. This nucleotide sequence was deposited in GenBank under accession no. HQ223415, and it showed 99% identity with previously deposited OvHV-2 sequences.

**Figure:**
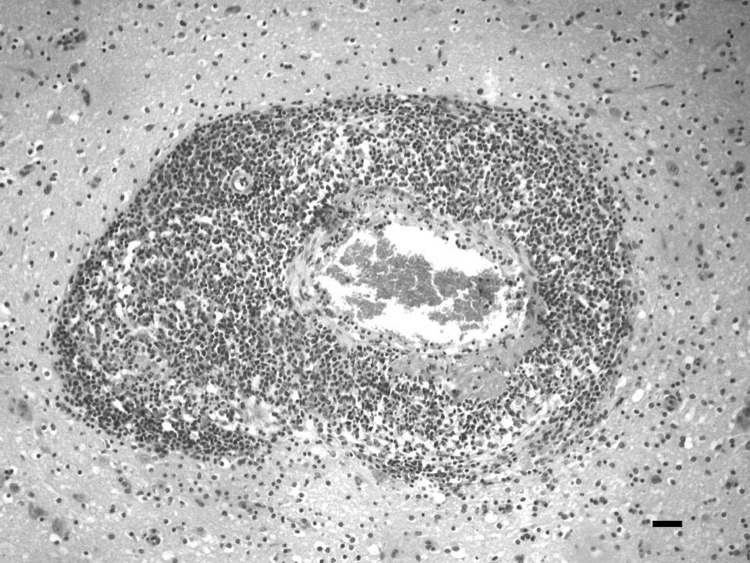
Brainstem of a sow with vasculitis that was associated with ovine herpesvirus 2, showing edema, necrosis (fibrinoid necrosis), and infiltration of lymphocytes in the adventitia of the artery. Severe perivascular (Virchow-Robin space) lymphocytic cuffing was observed. Hematoxylin and eosin stain; scale bar = 50 μm.

To find possible carriers of the virus, blood samples were collected from 9 pregnant sows, 10 nonpregnant sows, and 30 breeding boars and analyzed for OvHV-2 DNA. Samples from 3 boars were positive. Nasal swabs and semen samples were collected from these infected boars to investigate the potential mode of OvHV-2 transmission, and OvHV-2 DNA was detected only in semen samples. Two of the 3 semen samples had >350 copies/2 µg of total DNA, suggesting that these animals shed virus (*8**,**9*). During this period, all infected boars remained clinically healthy.

In Brazil, porcine MCF has been found primarily in pregnant sows and gilts. Our findings of OvHV-2 DNA in the semen of asymptomatic boars suggest that the OvHV-2 in the sows and gilts originated from asymptomatic boars that were responsible for maintaining the virus in the herd. Whether virus shedding in the semen was temporary or lasted for a long period is not known.

Emergence of OvHV-2 in boars that had no known contact with sheep was surprising, especially given the possibility of venereal transmission through contaminated semen. The occurrence of OvHV-2 infection in other specific pathogen–free farms is unknown, and it is not possible to suggest a strategy to guarantee OvHV-2-free herds.

## References

[1] Loken T, Aleksandersen M, Reid HW, Pow I. Malignant catarrhal fever caused by ovine herpesvirus-2 in pigs in Norway. Vet Rec. 1998;143:464–7. 10.1136/vr.143.17.464 9829302

[2] Loken T, Bosman A-M, Van Vuuren M. Infection with ovine herpesvirus 2 in Norwegian herds with a history of previous outbreaks of malignant catarrhal fever. J Vet Diagn Invest. 2009;21:257–61. 10.1177/104063870902100216 19286510

[3] Gauger PC, Patterson AR, Kim WI, Stecker KA. An outbreak of porcine malignant catarrhal fever in a farrow-to-finish swine farm in the United States. J. Swine Health Prod. 2010;18:244–8.

[4] Baxter SIF, Pow I, Bridgen A, Reid HW. PCR detection of the sheep-associated agent of malignant catarrhal fever. Arch Virol. 1993;132:145–59. 10.1007/BF01309849 8352654

[5] Mesquita RA, Anzai EK, Oliveira RN, Nunes FD. Evaluation of 3 methods of DNA extraction from paraffin-embedded material for the amplification of genomic DNA using PCR [in Portuguese]. Pesqui Odontol Bras. 2001;15:314–9. 11787320 10.1590/s1517-74912001000400008

[6] Van Devanter DR, Warremer P, Bennett L, Schultz ER, Coulter S, Garber RL, Detection and analysis of diverse herpesviral species by consensus primer PCR. J Clin Microbiol. 1996;34:1666–71. 8784566 10.1128/jcm.34.7.1666-1671.1996 PMC229091

[7] Ehlers B, Ulrich S, Goltz M. Detection of two novel porcine herpesvirus with high simularity to gammaherpesvirus. J Gen Virol. 1999;80:971–8. 10211967 10.1099/0022-1317-80-4-971

[8] Albini S, Zimmermann W, Neff F, Ehlers B, Hani H, Li H, Identification and quantification of ovine gammaherpesvirus 2 DNA in fresh and stored tissues of pigs with symptoms of porcine malignant catarrhal fever. J Clin Microbiol. 2003;41:900–4. 10.1128/JCM.41.2.900-904.2003 12574312 PMC149657

[9] Hüssy D, Janett F, Albini S, Staüber N, Thun R, Ackermann M. Analysis of the pathogenetic basis for shedding and transmission of ovine gamma herpesvirus 2. J Clin Microbiol. 2002;40:4700–4. 10.1128/JCM.40.12.4700-4704.2002 12454175 PMC154612

